# Predictors of non-primary auditory and vestibular symptom persistence following surgical repair of superior canal dehiscence syndrome

**DOI:** 10.3389/fneur.2024.1336627

**Published:** 2024-02-26

**Authors:** Liliya Benchetrit, Samantha Shave, Alejandro Garcia, Janice J. Chung, Krish Suresh, Daniel J. Lee

**Affiliations:** ^1^Department of Otolaryngology–Head and Neck Surgery, Massachusetts Eye and Ear and Harvard Medical School, Boston, MA, United States; ^2^Department of Otolaryngology–Head, and Neck Surgery, Boston University, Boston, MA, United States; ^3^Rutgers Robert Wood Johnson Medical School, New Brunswick, NJ, United States; ^4^Department of Otolaryngology–Head, and Neck Surgery, University of Iowa, Iowa City, IA, United States

**Keywords:** superior canal dehiscence syndrome, cVEMP, vestibular evoked myogenic potential, third window, surgery, middle fossa craniotomy, transmastoid, mastoidectomy

## Abstract

**Objective:**

Patients with superior canal dehiscence syndrome (SCDS) can present with a plethora of auditory and/or vestibular symptoms associated with a bony defect of the superior semicircular canal. While surgical repair is a reasonable option for patients with significant localizing symptoms, the degree of clinical improvement will vary among patients and poses challenges in outcome prediction. This study aims to assess the relationship between preoperative and postoperative symptoms and identify predictors of symptom persistence following repair.

**Study design:**

Retrospective chart review.

**Setting:**

Tertiary neurotology single-institution care center.

**Main outcome measures:**

The primary outcome was to determine the proportion of resolved and persistent primary (most bothersome) and non-primary audiologic and vestibular symptoms following SCD repair. Secondary outcomes included comparison of patient, operative and radiologic characteristics between patients with resolved vs. persistent symptoms. Standardized patient questionnaires including 11 auditory and 8 vestibular symptoms were administered to patients at their preoperative and follow-up visits. Patient pre- vs. postoperative survey results, demographic and clinical characteristics, operative characteristics, audiometric data and cervical vestibular evoked myogenic potential (cVEMP) thresholds were compared via univariate χ^2^ and multivariate binary logistic regression analyses between those patients reporting full postoperative resolution of symptoms and persistence of one or more symptoms. Radiologic computed tomography (CT) measurements of superior canal dehiscence (SCD) defect size, location, and laterality were also compared between these two groups.

**Results:**

Of 126 patients (132 ears) included in our study, 119 patients (90.2%) reported postoperative resolution (*n* = 82, 62.1%) or improvement (*n* = 37, 28.0%) of primary (most bothersome) symptoms, while 13 patients (9.8%) reported persistence of primary symptoms. The median (interquartile range) and range between surgery and questionnaire completion were 9 (4–28), 1–124 months, respectively. Analyzing all symptoms (primary and non-primary) 69 (52.3%) and 68 (51.1%) patients reported complete postoperative auditory and vestibular symptom resolution, respectively. The most likely persistent symptoms included imbalance (33/65/67, 50.8%), positional dizziness (7/20, 35.0%) and oscillopsia (44/15, 26.7%). Factors associated with persistent auditory symptoms included history of seizures (0% vs. 7.6%, *p* = 0.023), auditory chief complaint (50.0% vs. 70.5%), higher PTA (mean 19.6 vs. 25.1 dB, *p* = 0.043) and higher cervical vestibular evoked myogenic potential (cVEMP) thresholds at 1000 Hz (mean 66.5 vs. 71.4, *p* = 0.033). A migraine diagnosis (14.0% vs. 41.9% *p* < 0.010), bilateral radiologic SCD (17.5% vs. 38.1%, *p* = 0.034) and revision cases (0.0% vs. 14.0%, *p* = 0.002) were associated with persistent vestibular symptoms. Neither SCD defect size nor location were significantly associated with symptom persistence (P > 0.05).

**Conclusions:**

Surgical repair for SCDS offers meaningful reduction in the majority of auditory and vestibular symptoms. However, the persistence of certain, mostly non-primary, symptoms and the identification of potential associated factors including migraines, PTA thresholds, cVEMP threshold, bilateral SCD, and revision cases emphasize the importance of individualized patient counseling and management strategies.

## Introduction

Third window syndrome encompasses a set of vestibular and auditory signs and symptoms that arise when a pathological third mobile window is present in the bony labyrinth of the inner ear. One of the most thoroughly studied conditions within this category is known as superior canal dehiscence syndrome (SCDS). This syndrome is due to a bony defect of the superior semicircular canal (SSC). This “third window” disrupts the bony barrier between the SSC and middle cranial fossa, most commonly at the arcuate eminence and, in rare cases, between the descending limb of the SSC and superior petrosal sinus (1, 2). The morphology of this bony defect, clinical presentation, and diagnostic indicators varies widely across patients. This has presented many challenges in terms of both diagnosis and clinical decision making. Some SCDS patients present with severe vestibular and/or auditory symptoms while others are minimally symptomatic. Patients experiencing unremitting localizing symptoms are possible candidates for surgical repair while the majority of SCDS patients can be observed.

There are currently no known medical therapies that are suitable for SCDS aside from avoidance or reduction of triggers. Surgery is a safe and reasonable management option, but there is a lack of consensus on which technique is the most effective. There are three main surgical approaches for the repair of SCD described in the literature - middle cranial fossa (MCF) approach, transmastoid approach (TM) and, round window reinforcement or plugging through a transcanal approach. The MCF approach has been the predominant technique for surgical repair, but there has been recent debate on the efficacy of the TM approach compared to MCF approach. The MCF approach provides better visualization; however, it carries an increased risk for morbidity of craniotomy with brain retraction (3, 4). In comparison, where TM approach lacks in direct visualization of the defect, it does avoid the morbidity of a craniotomy (4, 5). Of note, a recent literature review demonstrated that the MCF approach for the repair of SCD is associated with greater symptom resolution when compared to the TM approach (6). The primary goal of surgical repair is to create a watertight seal at the site of the defect. This is often described in the literature as gentle “plugging” or sealing of the dehiscence, where a variety of different materials have been described to achieve a watertight repair with no clear consensus among studies regarding specific technique. In contrast, attempts to “resurface” the bony defect are associated with higher rates of symptom recurrence compared to plugging or sealing of the SCD (7). In addition, the rate and durability of symptom resolution is highly variable, making clinical counseling challenging (5, 8–15).

Although many studies show clinical improvement in most surgical patients, a formal analysis on persistent symptoms following operative repair is limited (16–19). Some research has suggested that mechanically induced symptoms (i.e., low-frequency conductive hearing loss, autophony, pulsatile tinnitus, and sound- and pressure-induced vertigo) tend to resolve more readily compared to headaches, chronic disequilibrium, and brain fog. An analysis of three studies that included 124 patients reported postoperative resolution of symptoms of autophony, pulsatile tinnitus and sound- and pressure-induced vertigo in the range of 73%−100%, compared to 63%−95% for general disequilibrium and aural fullness. What is not well understood are the preoperative variables that predict postoperative clinical outcomes (5, 16, 20–22).

Theories to explain residual symptoms include “unmasking” of the contralateral ear in patients with bilateral dehiscence, iatrogenic alteration of the vestibular system or comorbid pathologies, and limitations of modern surgical repair techniques. Several studies have shown worse outcomes in patients with bilateral disease compared to those with unilateral disease, and it has come into question whether patients with bilateral defects receive bilateral repair or only unilateral repair (19, 21, 23–26). In addition, our group identified that in female patients, three factors were associated with prolonged recovery: (1) a history of migraines, bilateral SCD, and a larger dehiscence. In the same study, we observed a predominance residual vestibular symptom compared to auditory symptoms, with ~37% of patients experiencing continued balance issues postoperatively (16). Migraines are an important comorbid condition associated with ongoing vestibular issues after repair (16, 27).

We will extend our prior observations to identify clinical features of SCD patients that report symptom persistence following operative repair and evaluate the relationship between preoperative diagnostic indicators and clinical outcomes to improve patient counseling and surgical decision making.

## Materials and methods

This retrospective cohort study comprised a review of patients diagnosed with SCDS for which they underwent a surgical repair at our institution between 2002 and 2021. This study was approved by the Institutional Review Board (IRB) at Massachusetts General Brigham under protocol number (2021P001712).

### Subjects, survey instruments, and surgical intervention

Patient data were obtained retrospectively from the electronic medical records (EMR) and managed using REDCap electronic data capture tools hosted at Massachusetts Eye and Ear Infirmary (28, 29). Patient (sex, age, past medical history, comorbid otologic/vestibular conditions, symptom duration) and surgical (radiologic laterality, surgical approach, endoscope use, and primary or revision case) characteristics were recorded.

Responses on a standardized symptomatology questionnaire collected from patients at their preoperative and postoperative visits were recorded. This questionnaire has been previously used successfully for research by our group (19, 30, 31). Briefly, the questionnaire asked patients to identify if their most bothersome complaint is hearing-related (auditory chief complaint) or balance-related (vestibular chief complaint). Binary replies (yes/no) were then recorded for the subjective experience of 11 auditory symptoms (hearing loss, aural fullness, pulsatile tinnitus, non-pulsatile tinnitus, autophony (hearing your voice too loudly), hyperacusis, hearing your voice echo, hearing your footsteps, hearing your eyeballs moving or hearing hair brushing or shaving sounds too loudly) and 8 vestibular symptoms (general dizziness, sense of imbalance, Tullio phenomenon, straining causing dizziness, physical activity causing dizziness, blowing your nose/sneezing/coughing causing dizziness, oscillopsia and positional dizziness). Information regarding postoperative resolution of primary (most bothersome) symptom complaint was obtained from the EMR and stratified to resolved, improved and persisted according to recorded patient description of symptoms.

Plugging of the SCD was performed through an MFC or TM approach. These surgical techniques have been described throughout the literature (2, 32). In summary, in the MFC approach the defect was plugged with bone wax, and then, any associated tegmen defects were repaired with temporalis fascia and split calvarial bone graft. When the TM approach was used, both limbs of the superior semicircular canal were plugged after labyrinthotomies were made on either side of the dehiscence with no direct contact with the defect (16). We excluded patients whose dehiscence was a result of tumor (cholesteatoma, epidermoid) extension given difficulty in ascertaining etiology of reported symptoms.

### Audiometric and VEMP data

Audiometric measurements were recorded from audiologic and vestibular testing during pre- and postoperative visits. Audiogram data included air- and bone- conduction thresholds. The bone conduction (BC) threshold was collected at 250, 500, 1, 000, 2000, and 4000 Hz pre- and postoperatively. The air-bone gap (ABG) was calculated at each tested frequency by subtracting the bone conduction threshold from the air conduction threshold. We collected ABG values at 250, 500, 1, 000, 2000, and 4000 Hz. Pure tone average (PTA) calculated by averaging air conduction thresholds at 500, 1000, and 2000 Hz were recorded. Lastly, cervical vestibular evoked myogenic potential (cVEMP) thresholds were obtained during sternocleidomastoid (SCM) contraction via four surface electromyography (EMG) electrodes.

Tone bursts were presented monaurally at a repetition rate of 13 bursts/second. To determine cVEMP thresholds responses were first obtained at 123 dB peSPL (equivalent to 90 dB HL) after which the sound level was decreased in 10 dB steps until no response could be distinguished from residual noise. To determine threshold, sound levels were then raised by 5 dB. Threshold was defined as the lowest sound level at which a cVEMP was present as determined by the audiologist performing the cVEMP. If no response was identified at the highest possible stimulus intensity (133 dB peSPL) cVEMP threshold was defined as 10 dB higher than our equipment limit, for this would have been the next sound level used. cVEMP thresholds at 500, 750, and 1000 Hz were collected and analyzed. This method has previously been used and described by our institution (33).

### Superior canal dehiscence defect size and location

**Figure 1:**
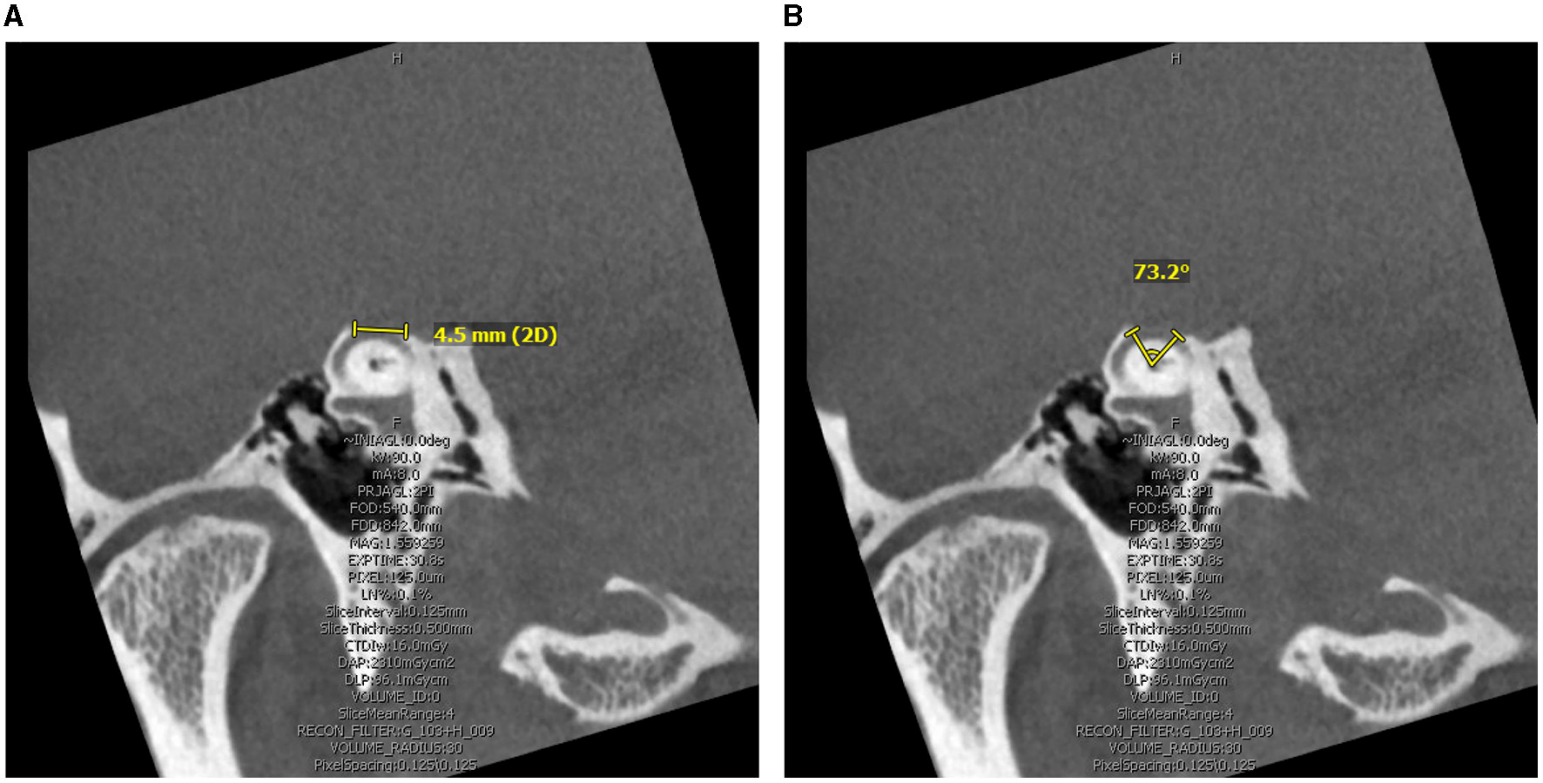
Computed Tomography superior canal dehiscence (SCD) measurement in the Pöschl view. **(A)** Linear length measurement obtained as the linear distance between the two ends of the SCD. **(B)** Arc length measurement obtained by radii and angle measure approximating defect center by the arcuate artery.

**Figure 2:**
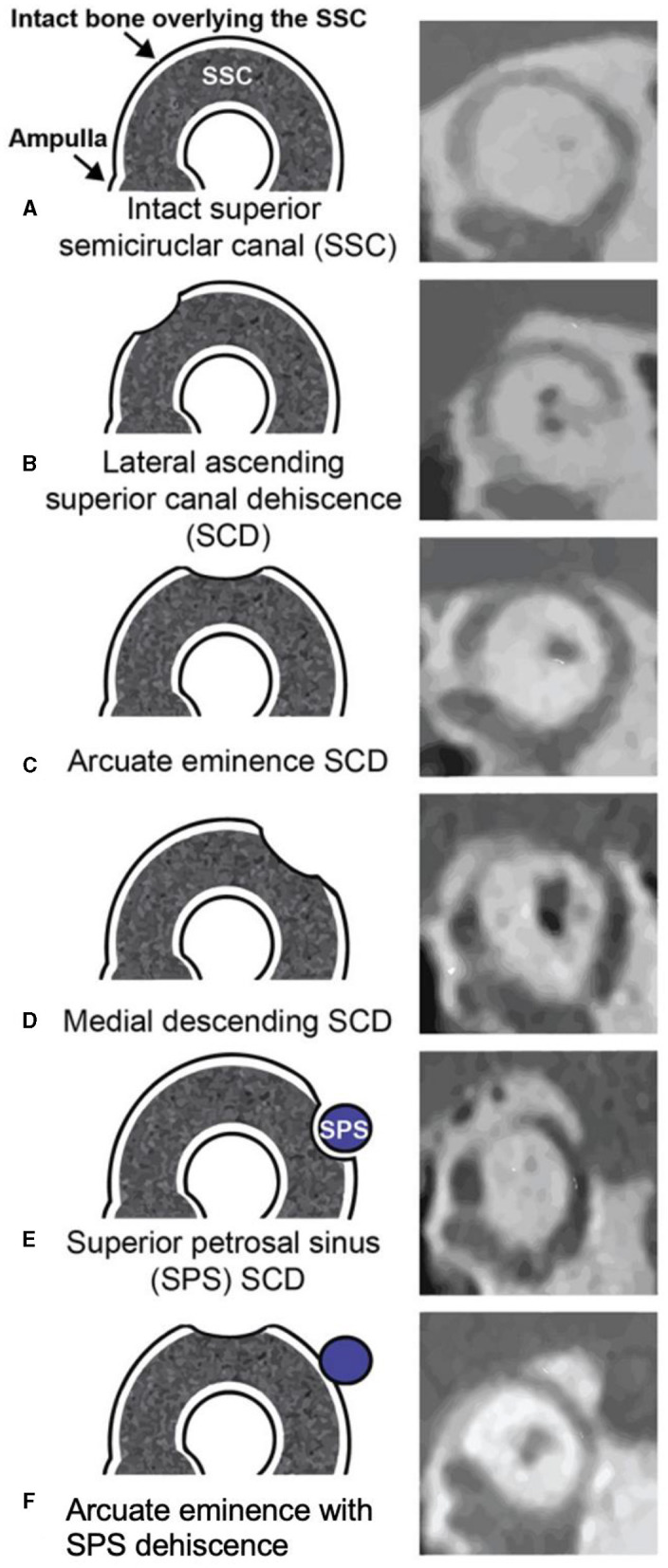
Obtained with permission from Lookabaugh et al. (34). Classification of superior canal dehiscence (SCD) based on location. Left column, diagram of defect location; right column, computed tomography images of corresponding defect location. **(A)** Intact superior semicircular canal (SSC), **(B)** Dehiscence on the lateral SSC upslope, **(C)** Dehiscence of the arcuate eminence, **(D)** Dehiscence on the medial SSC downslope, **(E)** Superior petrosal sinus-associated SCD (SPS-SCD), **(F)** Arcuate eminence and SPS dehiscence.

Two independent author raters (AG and JC) measured the size of the defect on pre-operative computed tomography (CT) using the same methods. The Pöschl view, which is perpendicular to the long axis of the temporal bone and parallel to the SSC plane, was analyzed for two measurements: chord length (linear distance between the two ends of the SCD) and arc length (two equal radii were measured from the center – approximated by the arcuate artery – to the two defect ends) (Figure 1). SCD location was described according to our institution's radiologic classification study and include: dehiscence of the lateral upslope (ascending limb of SCC), arcuate eminence, medial downslope (descending limb of SSC), proximity to superior petrosal sinus (SPS), and combined arcuate eminence and SPS defects, as shown in Figure 2 (34).

Our cohort had six patients who underwent bilateral sequential SCD repair surgery. We included each case separately with symptom questionnaire and objective testing obtained before and after each operated ear case.

### Statistical analysis

Baseline cohort characteristics were summarized as proportions. Continuous variables were reported by median and interquartile range (IQR) or mean and standard deviation (SD). Each patient's symptom response on the survey was analyzed pre- and postoperatively and classified postoperatively into (1) resolved (if patient endorsed the symptom preoperatively and denied it postoperatively), (2) persistent (if patient endorsed the symptom pre and post operatively), or (3) new symptom (if patient denied the symptom preoperatively but endorsed it post operatively). Primary (most bothersome) symptom status was classified into resolved, improved, and persisted. The McNemar's test for paired nominal data was used to compare the proportions of resolved and persistent symptoms as recorded from survey responses, and resolved, improved and persistent primary symptoms as recorded from the EMR. We evaluated the association between patient, audiometric/vestibular results and surgical/SCD characteristics with auditory and vestibular symptom status (resolved vs. persistent – persistent status was defined as at least one persistent symptom, regardless of whether reported as primary symptom or not) using univariate χ^2^ and Fisher exact (in cases of < 10 cases) test for categorical variables. An independent *t* test and the Mann–Whitney *U*-test were used to compare means of continuous variables with normal and non-normal distribution, respectively. Factors identified significant on univariate analyses were associated with auditory and vestibular symptom persistence status via multivariate binary logistic regression. Regression models were assessed for multicollinearity and variables with variance inflation factors >4 were removed (35). A Pearson correlation was used to evaluate the relationship between the mean number of symptoms a patient reported (stratified by auditory and vestibular) with SCD defect size and audiometric and vestibular test results, both pre- and post- operatively. Missing values were excluded. All data analyses were performed using SPSS version 29.0 (SPSS, Inc). Significance was determined at the *P* < 0.05 level.

## Results

### Patient characteristics and presenting signs and symptoms

Our institution manages a database of almost 800 pediatric and adult patients with SCD. We identified a total of 126 patients (132 ears) who had underwent surgical repair for SCDS and met inclusion criteria for this study. The median (IQR) age of patients at the time of surgery was 50.6 (16.7) years. Most of the patients were female (55.6%). The median (IQR) length of follow-up from surgery to time of postoperative questionnaire administration was 9.0 (24.0) months. Thirty-five (27.8%) patients had radiologic evidence of bilateral SCD. The mean (SD) arc and linear SCD length were 4.2 (1.9) mm and 3.9 (1.5) mm, respectively. Middle fossa craniotomy was performed in the majority (92.4%) of cases. The remainder of baseline characteristics is shown in Table 1. All patients underwent canal plugging. The number of audiologic and vestibular symptoms, ABG at 250 Hz, 500 Hz and 1000 Hz and all tested cVEMP thresholds showed significant postoperative improvement (*p* < 0.001) (Table 2). There was no significant difference in pre (22.2 dB) - vs. postoperative (21.5 dB) PTA (*p* = 0.726). However, BC thresholds increased significantly among all tested frequencies other than 2000 Hz (Table 2).

**Table 1:** Baseline cohort characteristics.

**Patient characteristics**	**All *N =* 132 cases (100%)**	*P-*value^*^
Sex		0.076
Male	56 (44.4%)	
Female	70 (55.6%)	
Age at surgery, median (IQR), y	50.6 (41.1–57.8)	
PMH DM	10 (7.9%)	< 0.001
PMH HTN	43 (34.1%)	< 0.001
PMH obesity (BMI > 30)	22 (17.4%)	< 0.001
BMI (kg/m^2^), median (IQR)	27.6 (24.9–31.7)	
PMH seizures	5 (4.0%)	< 0.001
Migraine diagnosis	40 (27.5%)	< 0.001
Prior ear surgery or infection	13 (10.6%)	< 0.001
Head Trauma	23 (18.3%)	< 0.001
Brain fog	13 (10.3%)	< 0.001
Symptom duration prior to SCD diagnosis, median (IQR), m	24.0 (8.8–60.0)	
Follow-up duration (surgery to questionnaire), median (IQR), range, m	10.0 (4.0–28.0), 1.0–124.0	
Chief complaint		
Auditory	76 (59.8%)	0.002
Vestibular	51 (40.2%)	
**SCD/operative characteristics**		
Radiologic laterality		0.025
Right	39 (30.9%)	
Left	52 (41.3%)	
Bilateral	35 (27.8%)	
SCD location		< 0.001
Lateral upslope	30 (22.7%)	
Arcuate eminence	79 (59.8%)	
Medial downslope	17 (12.9%)	
SPS	3 (2.3%)	
Arcuate + SPS	1 (0.8%)	
SCD arc length (mean, SD), mm	4.2 (1.9)	
SCD linear length (mean, SD), mm	3.9 (1.5)	
Surgical Approach		< 0.001
Middle fossa craniotomy	122 (92.4%)	
Transmastoid	10 (7.6%)	
Endoscope		< 0.001
Used	40 (30.3%)	
Not used	89 (67.4%)	
OSH revision case	8 (6.1%)	< 0.001
Underwent revision post index case	19 (14.4%)	< 0.001

^*^P-value represents comparison of proportions within each category via the χ^2^ or Fisher exact test (if n < 10). Categories with percentages not adding to 100% are due to unknown/missing information. Categories with only one measured group correlates to those with the disease/symptoms. y, years; m, months; IQR, interquartile range; DM, diabetes mellitus; HTN, hypertension; MBI, body mass index; SCD, superior canal dehiscence; SD, standard deviation; SPS, superior petrosal sinus; OSH, outside hospital.

**Table 2:** Symptom audiometric and cVEMP results pre- and post- operatively.

	**Preoperative**	**Postoperative**	*P*-value^*^
**Symptoms (mean, SD)**			
Number of auditory symptoms	3.8 (2.3)	1.2 (1.5)	**< 0.001**
Number of vestibular symptoms	2.9 (1.6)	1.2 (1.2)	**< 0.001**
**Audiometric and cVEMP testing (mean, SD)**			
ABG 250 Hz (dB)	23.8 (15.9)	9.1 (12.0)	**< 0.001**
ABG 500 Hz (dB)	16.1 (13.3)	6.8 (9.8)	**< 0.001**
ABG 1000 Hz (dB)	13.1 (11.6)	6.6 (8.7)	**< 0.001**
ABG 2000 Hz (dB)	3.9 (7.6)	2.8 (5.2)	0.181
ABG 4000 Hz (dB)	5.2 (8.1)	5.5 (7.5)	0.761
PTA dB	22.2 (14.9)	21.5 (16.7)	0.726
Bone conduction 250 Hz (dB)	2.9 (12.8)	10.2 (12.5)	**< 0.001**
Bone conduction 500 Hz (dB)	8.6 (12.8)	15.6 (13.5)	**< 0.001**
Bone conduction 1000 Hz (dB)	7.2 (12.9)	13.1 (14.1)	**< 0.001**
Bone conduction 2000 Hz (dB)	18.9 (15.9)	19.8 (18.1)	0.151
Bone conduction 4000 Hz (dB)	22.2 (19.4)	27.2 (20.3)	**< 0.001**
cVEMP 500 Hz (dB HL)	65.9 (12.3)	86.9 (10.6)	**< 0.001**
cVEMP 750 Hz (dB HL)	66.5 (12.1)	87.8 (10.6)	**< 0.001**
cVEMP 1000 Hz (dB HL)	68.9 (11.4)	89.1 (9.9)	**< 0.001**

^*^P-value for comparison of pre and post- operative means via two sample t-test. Bolded values with an ^*^ indicate a statistically significant result.

### Postoperative symptoms

**Figure 3:**
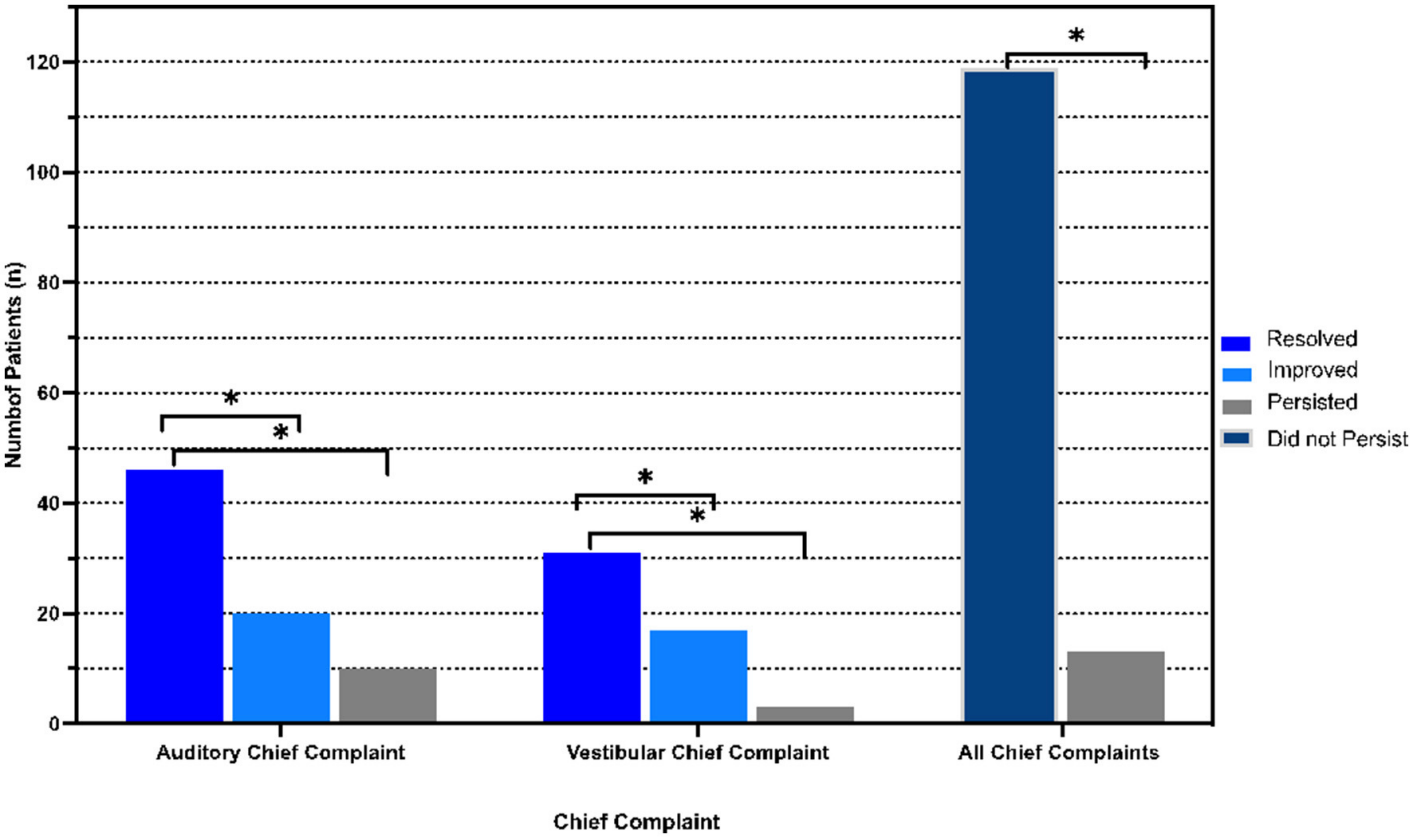
Postoperative distribution of primary symptoms. Resolved, improved, persistent and non-persistent (combination of resolved and improved) symptom distribution stratified by primary symptom (chief complaint). *p < 0.05 indicates statistical significance.

**Figure 4:**
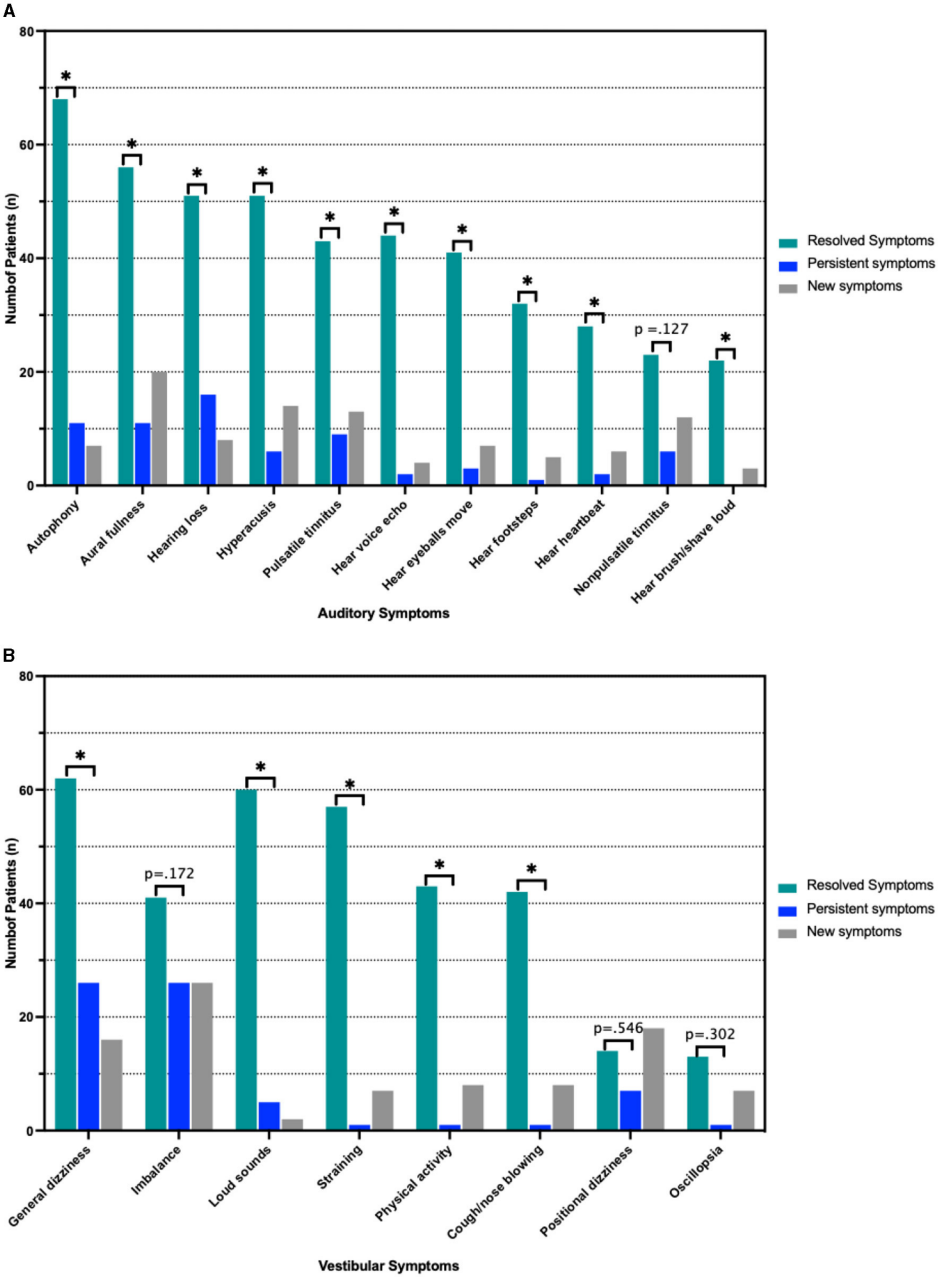
Pre- and Postoperative symptoms. **(A)** Resolved, persistent and new auditory symptoms according to number of patients reporting them. **(B)** Frequency of vestibular symptoms stratified as above. *p < 0.05 indicated statistical significance comparing resolved and persistent symptoms. Actual *p*-value is shown in cases with no statistically significant difference between resolved and persistent symptoms.

In this cohort, 119 patients (90.2%) reported postoperative resolution (*n* = 82, 62.1%) or improvement (*n* = 37, 28.0%) of primary symptoms, while 13 patients (9.8%) reported persistence of primary symptoms (*p* < 0.001 between persistent and non-persistent symptomatology). Of these 13 patients, 10 (76.9%) reported an auditory chief complaint and 3 (23.1%) patients reported a vestibular chief complaint, *p* = 0.007 (Figure 3). Analyzing all symptoms (primary and secondary), 63 (47.7%) and 64 (48.5%) patients reported persistence of at least one auditory symptom and one vestibular symptom, respectively. All auditory symptoms showed a statistically significant difference between proportion resolution and persistence (*p* < 0.001 for all) (Figure 4A, Supplementary Table 1). Persistent vestibular symptoms included a sense of imbalance (24.2% resolved vs. 25.0% persistent, *p* = 0.880), positional dizziness (9.8% resolved vs. 5.3% persistent, *p* = 0.167) and oscillopsia (8.3% resolved vs. 3.0% persistent, *p* = 0.063) (Figure 4B, Supplementary Table 1).

**Figure 5:**
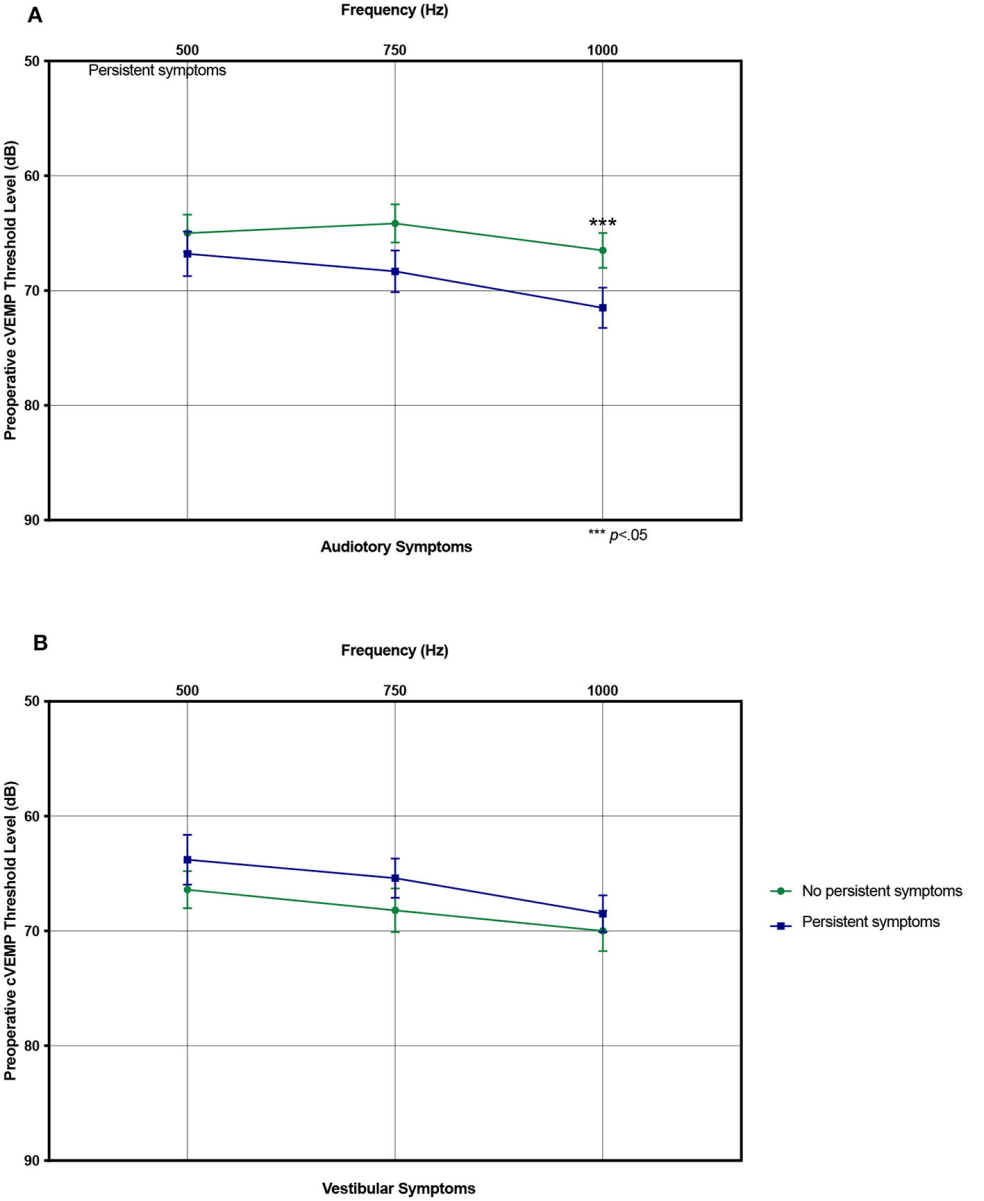
Preoperative cervical vestibular evoked myogenic potential (cVEMP) thresholds. **(A)** Mean preoperative cVEMP thresholds stratified by resolved and persistent postoperative auditory symptoms. **(B)** Same showing for vestibular symptoms. ***p < 0.05 indicating statistical significance comparing resolved and persistent symptoms. Error bars denote standard error.

Factors associated with persistent auditory symptoms included history of seizures (0% vs. 7.9%, *p* = 0.023), auditory chief complaint (50.0% vs. 75.0%), greater number of pre-operative auditory symptoms (3.1 vs. 4.7, *p* < 0.001), higher preoperative PTA threshold (19.6 vs. 25.1 dB, *p* = 0.043) and higher preoperative cVEMP thresholds at 1000Hz (66.5 vs. 71.4 dB HL, *p* = 0.033). Patients with persistent auditory symptoms were more likely to undergo revision surgery (7.4% vs. 22.2%) (Table 3, Figure 5). Factors associated with persistent vestibular symptoms included a migraine diagnosis (20.6% vs. 41.3% *p* < 0.001), number of pre-operative auditory (3.4 vs. 4.3, *p* = 0.040) and vestibular (2.6 vs. 3.3, *p* = 0.011) symptoms, bilateral radiologic SCD (17.5% vs. 38.1%, *p* = 0.034) and patients presenting for a revision case from an outside hospital (0.0% vs. 14.0%, *p* = 0.002) (Table 4). Neither SCD defect size nor location significantly associated with audiologic (Table 3) or vestibular (Table 4) symptom persistence (*p* > 0.05).

**Table 3:** Clinical and demographic characteristics by postoperative auditory symptom status.

	**Postoperative auditory symptom status**		***P*-value**
	**Resolved** ***N*** = **69 (52.3%)**	**Persistent (at least 1)** ***N*** = **63 (47.7%)**	**0.456**
**Patient characteristics**			
Sex			0.294
Male	33 (47.8%)	24 (38.1%)	
Female	36 (52.2%)	39 (61.9%)	
Age at surgery, median (y)	48.9 (36.8–58.2)	52.6 (46.4–57.8)	0.101
PMH DM	3 (4.3%)	7 (11.1%)	0.193
PMH HTN	24 (34.8%)	19 (30.2%)	0.583
PMH obesity	8 (11.6%)	14 (22.2%)	0.110
PMH seizures	0 (0.0%)	5 (7.9%)	**0.023** ^*^
Migraine diagnosis	19 (27.5%)	21 (33.9%)	0.432
Prior ear surgery or infection	4 (6.3%)	9 (14.5%)	0.152
Head Trauma	12 (19.0%)	11 (20.0%)	896
Brain fog	6 (9.4%)	7 (12.1%)	1.000
Symptom duration prior to SCD diagnosis (mean, SD), months	39.1 (51.9)	38.7 (40.9)	0.274
Duration of follow up (mean, SD), months	12.3 (97.7)	23.4 (29.2)	0.416
Chief complaint			**0.19**
Auditory	33 (50.0%)	43 (70.51%)	
Vestibular	23 (50.0%)	18 (29.5%)	
Number of preoperative auditory symptoms (mean, SD)	3.1 (2.2)	4.7 (2.1)	**< 0.001** ^*^
Number of preoperative vestibular symptoms (mean, SD)	2.8 (1.5)	3.1 (1.7)	0.322
**Audiologic characteristics (mean, SD)**			
Pre-op ABG 250 Hz (dB)	21.9 (14.6)	25.9 (17.0)	0.160
Pre-op ABG 500 Hz (dB)	16.4 (14.4)	15.8 (12.3)	0.810
Pre-op ABG 1000 Hz (dB)	12.6 (11.6)	13.5 (11.8)	0.669
Pre-op ABG 2000 Hz (dB)	3.8 (775)	4.1 (7.6)	0.804
Pre-op ABG 4000 Hz (dB)	5.4 (7.9)	5.2 (8.2)	0.569
Pre-op PTA dB	19.6 (12.8)	25.1 (16.7)	**0.043** ^*^
Pre-op cVEMP 500 Hz (dB HL)	65.0 (0.4)	66.8 (13.9)	0.487
Pre-op cVEMP 750 Hz (dB HL)	64.2 (10.7)	68.3 (12.9)	0.093
Pre-op cVEMP 1000 Hz (dB HL)	66.5 (9.8)	71.4 (12.5)	**0.033** ^*^
**SCD/operative characteristics**			
Radiologic Laterality			0.490
Right	22 (32.8%)	16 (27.1%)	
Left	29 (43.2%)	23 (39.0%)	
Bilateral	16 (23.8%)	20 (33.9%)	
SCD Location			0.226
Lateral upslope	14 (20.6%)	16 (25.8%)	
Arcuate eminence	42 (61.8%)	37 (59.7%)	
Medial downslope	11 (16.2%)	6 (9.7%)	
SPS	0 (0.0%)	3 (4.8%)	
Arcuate + SPS	1 (1.5%)	0 (0.0%)	
SCD arc length, mean (SD)	4.0 (1.9)	4.4 (1.9)	0.293
SCD linear length, mean (SD)	3.7 (1.6)	4.0 (1.5)	0.319
Surgical approach			0.193
Middle fossa craniotomy	66 (95.7%)	56 (88.9%)	
Transmastoid	3 (4.3%)	7 (22.2%)	
OSH Revision case	3 (4.6%)	5 (8.5%)	0.476
Underwent revision post index case	5 (7.4%)	14 (22.2%)	**0.024** ^*^

Bolded values with an * indicate a statistically significant result.

**Table 4:** Clinical and demographic characteristics by postoperative vestibular symptom status.

	**Postoperative vestibular symptom status**		***P*-value**
	**Resolved** ***N*** = **68 (51.5%)**	**Persistent (at least 1)** ***N*** = **64 (48.5%)**	
**Patient characteristics**			
Sex			0.823
Male	30 (44.1%)	27 (42.2%)	
Female	38 (55.9%)	37 (57.8%)	
Age at surgery, median (y)	52.4	50.6	0.370
PMH DM	5 (7.4%)	5 (7.8%)	1.000
PMH HTN	22 (32.4%)	21 (32.8%)	0.955
PMH obesity	12 (17.6%)	10 (15.6%)	0.755
PMH seizures	2 (2.9%)	3 (4.7%)	0.673
Migraine diagnosis	14 (20.6%)	26 (41.3%)	**0.010** ^*^
Prior ear surgery or infection	6 (9.2%)	7 (11.5%)	0.774
Head Trauma	12 (18.2%)	11 (21.2%)	0.686
Brain fog	4 (5.9%)	9 (16.7%)	0.076
Symptom duration prior to SCD diagnosis (mean, SD), months	40.4 (51.5)	37.2 (40.5)	0.764
Duration of follow up (mean, SD), months	22.1 (32.7)	13.4 (97.5)	0.263
Chief complaint			0.745
Auditory	38 (58.5%)	38 (61.3%)	
Vestibular	27 (41.5%)	24 (38.7%)	
Number of preoperative auditory symptoms (mean, SD)	3.4 (2.0)	4.3 (2.5)	**0.040** ^*^
Number of preoperative vestibular symptoms (mean, SD)	2.6 (1.5)	3.3 (1.7)	**0.011** ^*^
Pre-op ABG 250 Hz (dB)	25.9 (17.6)	21.6(13.6)	0.141
Pre-op ABG 500 Hz (dB)	17.7 (14.2)	15.0 (12.3)	0.215
Pre-op ABG 1000 Hz (dB)	13.3 (12.7)	12.8 (10.6)	0.825
Pre-op ABG 2000 Hz (dB)	4.4 (9.5)	3.5 (4.7)	0.521
Pre-op ABG 4000 Hz (dB)	5.9 (9.5)	4.6 (6.1)	0.350
Pre-op PTA dB	23.8 (15.3)	20.5 (14.6)	0.231
Pre-op cVEMP 500 Hz (dB HL)	64.5 (11.2)	67.6(13.4)	0.234
Pre-op cVEMP 750 Hz (dB HL)	65.7 (11.5)	67.2 (12.9)	0.562
Pre-op cVEMP 1000 Hz (dB HL)	67.2 (12.9)	70.6 (12.5)	0.167
**SCD/operative characteristics**			
Radiologic Laterality			**0.034** ^*^
Right	23 (36.5%)	16 (25.4%)	
Left	29 (46.0%)	23 (36.5%)	
Bilateral	11 (17.5%)	24 (38.1%)	
SCD Location			**0.612**
Lateral upslope	17 (25.8%)	13 (20.3%)	
Arcuate eminence	37 (56.1%)	42 (65.6%)	
Medial downslope	10 (15.2%)	7 (10.9%)	
SPS	1 (1.5%)	2 (3.1%)	
Arcuate+SPS	1 (1.5%)	0 (0.0%)	
SCD arc length, mean (SD)	4.4 (1.9)	3.9 (1.7)	0.177
SCD linear length, mean (SD)	4.1 (1.6)	3.7 (1.5)	0.180
Surgical Approach			0.197
Middle fossa craniotomy	65 (95.6%)	57 (89.1%)	
Transmastoid	3 (4.4%)	7 (10.9%)	
OSH Revision case	0 (0.0%)	8 (14.0%)	**0.002** ^*^
Underwent revision post index case	8 (11.9%)	11 (17.2%)	0.461

Bolded values with an * indicate a statistically significant result.

On multivariate binary logistic regression analysis evaluating the factors found significant on univariate analysis (excluding number of pre-operative auditory symptoms due to multicollinearity) and clinically deemed relevant factors, higher preoperative cVEMP thresholds at 1000 Hz were associated with a higher risk of auditory symptom persistence [Odds Ratio (OR): 1.05, 95% Confidence Interval (CI): 1.01–1.10] (Table 5). For vestibular symptoms, multivariate analysis (excluding number of preoperative vestibular symptoms variable due to multicollinearity) showed association of migraines (OR: 2.5, 95% CI: 1.19–7.96) and bilateral radiologic SCD (OR: 3.08, 95% CI: 1.24–7.65) with risk of symptom persistence (Table 6).

**Table 5:** Patient characteristics associated with postoperative persistent auditory symptoms on multivariable binary logistic regression^**^.

	**Risk of persistent auditory symptoms OR (95% CI)**	***P*-value**
PMH seizures		0.999
No	*1 (Reference)*	
Yes	~14^8^ (0.00–∞)	
Chief complaint		0.063
Auditory	*1 (Reference)*	
Vestibular	2.63 (0.95- 7.30)	
SCD laterality		0.828
Unilateral	*1 (Reference)*	
Bilateral	1.14 (0.36–3.63)	
Preop PTA dB	1.03 (0.99–1.07)	0.154
Preop cVEMP 1000 Hz	1.05 (1.01–1.10)	**0.028** ^*^

OR, odds ratio; CI, confidence interval; PMH, past medical history; ABG, air-bone gap; SCD, superior canal dehiscence. Preoperative PTA and cVEMP were continuous variables with OR results referring to ascending (higher) values. ^**^Variables found significant on univariate analysis and clinically relevant variables included. Bolded values with an * indicate a statistically significant result.

**Table 6:** Patient characteristics associated with postoperative persistent vestibular symptoms on multivariable binary logistic regression^**^.

	**Risk of persistent vestibular symptoms OR (95% CI)**	***P*-value**
Migraines		**0.039** ^*^
No	*1 (Reference)*	
Yes	2.50 (1.19–7.96)	
Laterality		
Unilateral	*1 (Reference)*	**0.016** ^*^
Bilateral	3.08 (1.24–7.65)	
Number of preoperative auditory symptoms	1.14 (0.96–1.35)	0.144
OSH revision case		
No	*1 (Reference)*	
Yes	~14^9^ (0.00–∞)	0.999

^**^Variables found significant on univariate analysis and clinically relevant variables are included. Bolded values with an * indicate a statistically significant result.

Interestingly, postoperative audiometric testing results were not significantly associated with risk of audiologic or vestibular symptom persistence (*p* > 0.05 for all) (Supplementary Table 2). Similarly, the number of vestibular and audiologic symptoms did not show significant correlation with audiometric testing, neither pre- nor postoperatively (*p* > 0.05 for all) (Supplementary Table 3).

## Discussion

Our study demonstrates significant improvement and resolution in the most bothersome SCDS symptoms as determined from record review and an overall significant reduction of both auditory and vestibular symptoms following surgical repair. We used a standardized questionnaire evaluating 11 auditory and 8 vestibular symptoms. A handful of persistent (majorly non-primary) symptoms and several associated preoperative factors such as migraines, PTA thresholds, cVEMP thresholds, bilateral SCD and revision cases were also identified. These findings highlight the nuances in the variable symptomatology and offer potential insight to preoperative counseling and postoperative recovery management. There are several proposed theories for why symptoms persist for patients following surgical repair. Many explanations include iatrogenic alteration of the vestibular system, psychosocial discrepancy in distinguishing prolonged recovery from persistent and/or new symptoms, the heightening of symptoms in the contralateral ear in cases of bilateral defects, etc. The vestibular system is part of a multisensory system structure, and direct surgical repair by “plugging” or resurfacing” can influence underlying function. Some studies suggest that plugging of the superior canal can lead to long-term deficits of the vestibulo-ocular reflex in response to head impulses (17, 36, 37). However, a recent study found that in response to the Subjective Visual Vertical test was not sensitive for identifying pathology after SCDS repair. This finding suggests that function may be well preserved following surgical plugging, supported by lowered, but still existent, VEMP responses in most patients (38). It is possible that patients may confuse prolonged surgical recovery with persistent or new symptoms related to their condition. The differentiation between symptom persistence and prolonged postoperative recovery is unclear, with no definitive time frame criteria or symptom severity criteria to distinguish between the two. One study with a cohort of 33 patients described clinical factors that are associated with prolonged recovery (average follow-up of 28.7 months) and reported that patients with bilateral SCD, a history of migraines, and larger defects may be at risk of prolonged recovery, however, this study was limited due to its small sample size (16). Our study had a median follow-up time of 9 months (IQR 9–28), range 1–124 months but with a notably larger cohort of 126 patients. This study thus aims to expand our understanding of the factors associated with symptom persistence using a much larger cohort in an effort to (1) improve clinical counseling and patient expectations following surgical repair (2) potentially define the difference between persistent symptoms and prolonged recovery, and (3) emphasize the importance of developing standardized methods for monitoring patient disease and outcomes using audiometric data, cVEMP testing, radiologic measurements, and a validated patient survey.

### Persistent and new postoperative symptoms

With regards to persistent auditory symptoms, our cohort followed a different pattern of audiometric findings compared to previous studies that describe persistent hearing loss postoperatively (11, 12, 39). Persistent mild SNHL following primary surgical repair of dehiscence, often in the form of high frequency hearing loss, is not uncommon (9, 40). In our study, there was a significant improvement in ABG at 250 Hz, 500 Hz, and 1000 Hz with no significant changes in the higher frequencies and there was significant resolution of all 11 subjective auditory symptoms, perhaps pointing to the comprehensive nature of the used survey.

Acute vestibular postoperative impairment is frequently reported in the literature. However, long-term persistence of this group of symptoms is limited. Additionally, acute vestibular impairment can occur after SCD repair, but most patients are able to compensate within several months (38, 41). This is consistent with vestibular impairment in the acute postoperative setting. Reduced SSC function alone can likely not explain cases of prolonged vestibular impairment. In our study patients with persistent vestibular symptoms after surgery presented mostly with a sense of imbalance, positional dizziness and oscillopsia with a prevalence below 20 %. For instance, prolonged vestibular impairment is common among patients with a concomitant migraine diagnosis or with bilateral SCDS, likely due to the more generalized vestibular impairment prior to surgery and a reduced ability of central compensation (16, 27). Previous studies have shown that patients with concurrent SCDS and migraine may have prolonged recovery after surgery (16, 27). The finding of bilateral radiologic dehiscence showing more persistent symptoms following a unilateral repair also supports the theory of “unmasking” by which symptoms might become more noticeable or be perceived as originating more clearly from the unoperated ear (8, 42). Bilateral dehiscence is an important finding in the preoperative workup, and it is critical that patients be counseled that they may be at higher risk for persistent, new, or heightened symptoms following surgery. Our findings demonstrated that patients with persistent vestibular symptoms presented with higher prevalence of vestibular migraine, bilateral radiologic superior canal dehiscence and were more likely to have been revision cases from other institutions.

### Size and defect location

There have been several studies that relate size and location of the bony defect with clinical presentation, disease severity, and audiometric data, but with no clear consensus (43, 44). Defect size is an important factor for surgical repair, as there may be a relationship between dehiscence size with hearing thresholds and symptom presentation (43). A variety of methods have been used to measure the size of the defect including three-dimensional curved reconstruction, measurement of the bone density profile, linear distance between identified ends of the defect, intraoperative manual measurements, and using number of radial sections in which the dehiscence was found to calculate the area of the defect (21, 34). The cohort described in this study demonstrated no association between defect size and location with persistence of symptoms, which is consistent with some studies but inconsistent with others. Our study was limited by a less comprehensive analysis of defect size, and further studies should aim to standardize defect measurement to best correlate this with patient presentation and surgical recovery.

### Body mass index and superior canal dehiscence syndrome

Due to prior research we were interested in the evaluation of persistent SCDS symptomatology with elevated BMI; however an association was not identified in our study (45, 46). Prior research has generally shown association of elevated BMI with sensorineural hearing loss (SNHL), and in the context of skull base defects, elevated BMI has been used as a surrogate indicator of elevated intracranial pressure (47, 48). The research regarding the relationship between obesity and SCDS is conflicting and still underway. One study found an increased incidence of obesity among patients with SCDS within a cohort of 31 patients; however, obesity was also found to not be related with symptomatic SCD (45, 49). This study also found no difference in incidence of BMI between surgical and non-surgical SCDS cases, and size of dehiscence poorly correlated with BMI. Theories regarding obesity and SCDS described that obesity has been shown to relate to increased intracranial pressure, thought to be due to increased intrathoracic pressure as a result of increased abdominal girth, thereby restricting cerebral venous drainage (50). The increased intracranial hypertension is hypothesized to lead to erosion of the skull base from direct repeated pulsations of the dura over time (51). With regards to SCDS, this erosion may thin the bone over the superior canal, thereby causing symptoms related to SCDS (46). While our study did not identify a significant difference, we had a relatively small cohort of patients with elevated BMI (17.5%, *n* = 22), and further research will assist to confirm or dispute the association in order to improve patient counseling.

### Audiometric and cVEMP data

Pure tone air and bone conduction threshold (including supranormal thresholds at −5 and −10 db) testing is routinely used for diagnosis as well as to monitor postoperative hearing. Primarily low frequency ABG is typically seen on diagnosis and postoperative closure of this gap indicates adequate SCD repair. Resolution of reduced hearing symptom has also been well associated with postoperative closure of the ABG (39). Some studies also describe that a larger ABG is associated with a larger defect, making audiometric data an important diagnostic tool in congruence with radiologic testing (43). In our study, ABG magnitude was not associated with postoperative auditory or vestibular symptom persistence (Tables 3, 4) and was also not associated with the number of presenting symptoms (Supplementary Table 2). Prior studies showed similar findings (31). These further highlight the discrepancy between diagnostic SCD testing and subjective patient experience. While the low-frequency ABG decreased significantly postoperatively, PTA thresholds remained stable. These findings were elucidated by bone conduction thresholds increasing significantly in the postoperative period. Given the paradoxical reduction in BC thresholds among patients with SCD, explained by hypersensitivity to vibrations (such as the stimulus used for BC) transmitted in the body, the corresponding increase in BC thresholds postoperatively is an additional indication of adequate SCD repair (52–54).

The mean PTA however, was significantly higher among patients with persistent auditory symptoms (19.6 dB) compared to those with resolved auditory symptoms (25.1 dB). While statistically significant, these PTA values may not represent true clinical significance given the overall small difference. Nevertheless, higher preoperative PTA values, measuring air conduction, may indicate an overall worse SCD-associated hearing loss. PTA has been associated with dehiscence size and postoperative speech discrimination scores, but these results are often not significant (55, 56). Clinically, PTA is a frequently used diagnostic tool for SCDS. Research is limited with regards to analyzing the relationship between persistent auditory symptoms and pre-operative PTA; therefore, future studies should investigate this variable.

VEMP testing is an important diagnostic indicator the evaluation of patients whom SCDS is suspected. Compared with healthy subjects, SCDS patients typically show lower cVEMP thresholds, higher cVEMP amplitudes, and air-bone gaps (ABG) (8, 57–62). Incongruously, our results showed higher (more normal) cVEMP thresholds at 1000Hz were associated with persistence of auditory symptoms. Hypothesizing that perhaps patients with more normal cVEMP thresholds were more likely to reports a primary auditory vs. vestibular chief complaint and as such symptom severity was not correlated with cVEMP thresholds, a sub-group analysis was conducted, but refuted this hypothesis (pre-operative cVEMP 1000 Hz thresholds mean of 67.2 dB HL for patients with auditory chief complaint vs. 72.3 dB HL with vestibular chief complaint, *p* = 0.069). Another explanation to this counterintuitive finding is that patients with higher cVEMP thresholds might have less severe or a smaller dehiscence, leading to subtler clinical symptoms (43). These subtler symptoms could be less amenable to surgical resolution, resulting in persistent postoperative auditory symptoms. Alternatively, patients with higher preoperative thresholds may have developed more robust central compensatory mechanisms to cope with vestibular dysfunction (8, 63). These compensatory pathways may continue to generate symptoms even after the anatomical defect has been corrected (64). Nevertheless, an association between elevated cVEMP thresholds and persistent auditory symptoms post-SCD repair challenges our traditional understanding of the condition. Other literature correlating VEMP data with symptom persistence is limited. Efforts made to improve the efficacy of cVEMP testing have shown that 2, 000-Hz tone bursts improve the detection of SCD, however, these studies have not been systematically applied during patient follow-up (65). These underscore the need for a comprehensive, nuanced approach when interpreting diagnostic tests and evaluating patient symptoms. It also emphasizes the need for future studies to focus on a deeper understanding of the interplay between objective tests and symptomatology to optimize diagnostic accuracy and postoperative care.

### Revision surgeries

In our cohort, patients that underwent revision surgery were more likely to experience persistent postoperative vestibular symptoms. Similar findings of less favorable outcomes with revision SCD surgery have been reported. Mozaffari et al. (66) studied 20 patients who underwent revision SCD repair and reported persistent symptoms of vertigo (67%), aural fullness (60%) and dizziness. Sharon et al. (67) evaluated 23 revision cases out of which only 35% of patients reported complete symptom resolution, as compared to ~66% of patients (out of 33) undergoing primary repair (16). Possible explanations involve an added surgical trauma to the inner ear labyrinth or neural structures from repeating surgeries and scar tissue from initial surgery increasing revision surgery associated trauma (67, 68). Additionally, it is possible that patients selected for revision surgeries represent a subset with more severe or complex SCDS, which could inherently be associated with a higher rate of symptom persistence. Consideration of the above results is imperative for comprehensive patient evaluation and counseling prior to revision surgery.

### Patient questionnaires

Similar to prior studies conducted by our institution, symptom reporting was conducted via a questionnaire administered by a clinician. Self-reporting systems are susceptible to significant bias. SCDS is an exceptionally variable condition, making clinical assessment of symptoms very challenging for clinicians. Some attempts have been made to standardize questionnaires. A recent standardized survey was developed in 2018, the Gopen-Yang Superior Semicircular Canal Dehiscence Questionnaire, which includes sections on general quality of life, internal amplified sounds, dizziness and tinnitus, with scores of 0–100 points (69). It is described as a holistic, patient-centered self-assessment; however, it was only used preoperatively. Our survey, although similar in content and focus, was used both pre- and postoperatively in order to provide insight into patient recovery. Validated questionnaires that have been previously used to quantify symptom presentation and resolution include the Autophony Index Dizziness Handicap Inventory (AIDHI), Hearing Handicap Inventory (HHI) and Tinnitus Handicap Inventory (70–73). However, these surveys were designed with the focus of specific symptoms, not a specific etiology. The Dizziness Handicap Inventory (DHI) and Headache Impact Test (HIT-6), were recently described as methods for assessing pre- and postoperative symptoms following surgical repair of cochlea-facial nerve dehiscence (74). A 2017 systematic review by Naert et al. (75) of 66 articles involving 431 patients with SCDS aggregated the 22 most commonly reported preoperative symptoms to be used as a basis for creation of a validated outcome-measure. Our questionnaire included 19 symptoms (11 auditory and 8 vestibular symptoms) with about 14 overlapping symptoms with Naert's 22-item list. No additional validated SCD patient-reported outcome measures have been described and the overall use of standard surveys for patients with diagnosed SCDS is limited. The inconsistent correlation between objective measures including audiometric and cVEMP data and SCD size and location with patient-reported symptoms in our study as well as past studies is noteworthy (31, 55). This further highlights the need for a robust standardized validated survey creating a patient-reported outcome measure to be used throughout clinical and/or surgical management which would allow for a more consistent and objective analysis of this highly subjective and variable condition. A more standardized and comprehensive system for measuring symptoms may improve symptom tracking, progression, and improvement following surgery.

### Limitations

There are noteworthy strengths and limitations for our study. First, this is one of the larger surgical SCDS cohorts evaluated both preoperatively and perhaps even more importantly postoperative allowing with both objective testing and symptom questionnaires. As discussed previously, patient symptoms were collected using a comprehensive but not yet validated questionnaire with a binary (yes/no) response limiting evaluation of symptom severity. The survey did not specifically include a question regarding postoperative resolution of chief complaint and as such, this was information we gathered from the EMR which could have been subject to a greater variability of interpretation. A more holistic representation of patient experience could be captured with a much-needed patient-reported outcome measure including additional quality-of-life assessment and grading of symptom severity. Given that only 13 patients reported postoperative persistence of their primary symptom which would subject analyses to low power bias, we focused the analyses on all persistent symptoms (which given the above were largely secondary). Furthermore, as some patients described more than one primary symptom, a separate primary symptom persistence analysis was not included. Additionally, the time-distinction between symptom persistence and prolonged postoperative recovery is not well defined in the literature, and our study (with a median follow up of 9 months and IQR 24.0 months) may have captured some prolonged recovery symptoms that could have resolved with a longer follow-up. Nevertheless, these findings can still be used for patient counseling. While only higher cVEMP thresholds, history of migraines and bilateral radiologic SCD continued to show a statistically significant association with secondary symptom persistence, we reported on and discussed history of revision cases, elevated BMI and seizures found significant only on univariate analysis due to their clinical significance and relevance gleaned from other studies. Reduced sample size available for the multivariate analysis (due to missing information) could have also contributed to lack of carried statistical significance. Additionally, symptom persistence can be partially in setting of a contralateral thin (though not dehiscent) SSC which was not a variable we were able to control for. We included multiple statistical analyses which could have increased the probability of false positive findings. The different analyses were pursued in order to thoroughly investigate and present the comprehensive variables our dataset captured.

Finally, our research was subject to all the inherent limitations of a retrospective cohort study and is potentially less generalizable given its single-center population of surgeons and patients.

## Conclusion

Although surgical repair has been shown to be highly effective and safe in the management of SCDS patients, our study shows that the chance of persistent or even new symptoms following surgery is an important consideration when counseling patients considering operative management. There are many limits to assessing how well a patient may respond to surgery including non-standardized radiologic evaluation, highly variable presenting symptoms and severity, as well as non-standardized clinical evaluation. The persistence of certain symptoms and the identification of potential associated factors including migraines, PTA thresholds, cVEMP thresholds, bilateral SCD and revision cases emphasize the importance of individualized patient counseling and management strategies. Despite the extensive literature on a variety of diagnostic testing, radiologic assessment, clinical presentation and surgical outcomes, there is a lack of consensus across centers. Future research should focus on prospective disease-specific validated patient symptom and quality-of-life survey data collection as a means to create standardized patient-reported outcome measures.

## Data availability statement

The raw data supporting the conclusions of this article will be made available by the authors, without undue reservation.

## Ethics statement

The studies involving humans were approved by the Massachusetts General Brigham Institutional Review Board. The studies were conducted in accordance with the local legislation and institutional requirements. Written informed consent for participation was not required from the participants or the participants' legal guardians/next of kin in accordance with the national legislation and institutional requirements.

## Author contributions

LB: Conceptualization, Data curation, Formal analysis, Investigation, Methodology, Writing – original draft, Writing – review & editing. SS: Conceptualization, Data curation, Formal analysis, Investigation, Methodology, Writing – original draft, Writing – review & editing. AG: Data curation, Writing – review & editing. JC: Data curation, Formal analysis, Methodology, Writing – review & editing. KS: Data curation, Writing – review & editing. DL: Conceptualization, Methodology, Writing – review & editing.
