# Supplementary material for: Predictors of non-primary auditory and vestibular symptom persistence following surgical repair of superior canal dehiscence syndrome
Source: Front Neurol. 2024 Feb 26;15:1336627. doi: 10.3389/fneur.2024.1336627 (PMC10925929; doi:10.3389/fneur.2024.1336627)
Supplement: Supplementary file 1 [file Data_Sheet_1.docx]

**SUPPLEMENTS**

| **Supplements Table 1. Likely in supplements (raw data for figure 1) Frequency of symptoms** | | | | | | |
| --- | --- | --- | --- | --- | --- | --- |
|  | **Preoperative**  **Symptom**    **N (%)** | **Resolved**  **Postoperative Symptom N (%)** | **Persistent**  **Postoperative Symptom** | **New**  **Postoperative**  **Symptom** | **No preoperative**  **Symptom** | **P-value*** |
| **Auditory** |  |  |  |  |  |  |
| Autophony | 79 (57.2%) | 68 (49.3%) | 11 (8.0%) | 7 (5.1%) | 52 (37.7%) | <.001 |
| Aural Fullness | 67 (48.6%) | 56 (40.6%) | 11 (8.0%) | 20 (14.5%) | 51 (37.0%) | <.001 |
| Hearing loss | 67 (48.6%) | 51 (37.0%) | 16 (11.6%) | 8 (5.8%) | 63 (45.7%) | <.001 |
| Hyperacusis | 57 (41.3%) | 51 (37.0%) | 6 (4.3%) | 14 (10.1%) | 67 (48.6%) | <.001 |
| Pulsatile tinnitus | 52 (37.7%) | 43 (31.2%) | 9 (6.5%) | 13 (9.4%) | 73 (52.9%) | <.001 |
| Hearing voice echo | 46 (33.3%) | 44 (31.9%) | 2 (1.4%) | 4 (2.9%) | 88 (63.8%) | <.001 |
| Hearing eyeballs move | 44 (31.9%) | 41 (29.7%) | 3 (2.2%) | 7 (5.1%) | 87 (63.0%) | <.001 |
| Hearing footsteps | 33 (23.9%) | 32 (23.2%) | 1 (0.7%) | 5 (3.6%) | 100 (72.5%) | .005 |
| Hearing heart beat | 30 (21.7%) | 28 (20.3%) | 2 (1.4%) | 6 (4.3%) | 102 (73.9%) | .019 |
| Nonpulsatile tinnitus | 29 (21.0%) | 23 (16.7%) | 6 (4.3%) | 12 (8.7%) | 97 (70.3%) | **.127** |
| Hearing brushing or shaving too loud | 22 (15.9%) | 22 (15.9%) | 0 (0.0%) | 3 (2.2%) | 113 (81.9%) | .054 |
| **Vestibular** |  |  |  |  |  |  |
| General Dizziness | 88 (63.8%) | 62 (44.9%) | 26 (18.8%) | 16 (11.6%) | 34 (24.6%) | <.001 |
| Sense of imbalance | 67 (48.6%) | 41 (29.7%) | 26 (18.8%) | 26 (18.8%) | 45 (32.6%) | **.172** |
| Loud sounds | 65 (47.1%) | 60 (43.5%) | 5 (3.6%) | 2 (1.4%) | 71 (51.4%) | <.001 |
| Straining | 58 (42.0%) | 57 (41.3%) | 1 (0.7%) | 7 (5.1%) | 73 (52.9%) | <.001 |
| Physical activity | 44 (31.9%) | 43 (31.2%) | 1 (0.7%) | 8 (5.8%) | 86 (62.3%) | <.001 |
| Cough or nose blowing | 43 (31.2%) | 42 (30.4%) | 1 (0.7%) | 8 (5.8%) | 87 (63.0%) | <.001 |
| Positional dizziness | 21 (15.2%) | 14 (10.1%) | 7 (5.1%) | 18 (13.0%) | 99 (71.7%) | **.546** |
| Oscillopsia | 14 (10.1%) | 13 (9.4%) | 1 (0.7%) | 7 (5.1%) | 117 (84.8%) | **.302** |
| **P-value comparing proportions of resolved versus persistent symptoms* | | | | | | |

| **Supplement Table 2. Association of postoperative audiometric results with persistence of symptoms** | | | | |
| --- | --- | --- | --- | --- |
|  | **Persistence of auditory symptoms**  **OR (95% CI)** | **P-value** | **Persistence of vestibular symptoms**  **OR (95% CI)** | **P-value** |
| Postoperative Audiometric Testing |  |  |  |  |
| ABG 250 Hz | 0.98 (0.95-1.01) | .183 | 1.02 (0.99-1.05) | .269 |
| ABG 500 Hz | 0.97 (0.95-1.02) | .430 | 1.00 (0.97-1.04) | .919 |
| ABG 1000 Hz | 1.00 (0.97-1.04) | .976 | 0.98 (0.94-1.01) | .223 |
| ABG 2000 Hz | 1.02 (0.96-1.08) | .546 | 1.01 (0.95-1.06) | .866 |
| ABG 4000 Hz | 1.02 (0.98-1.07) | .295 | 0.99 (0.95-1.03) | .610 |
| PTA dB | 1.02 (0.99-1.04) | .132 | 0.99 (0.97-1.02) | .559 |
| cVEMP 500 Hz | 0.97 (0.93-1.01) | .101 | 1.02 (0.98-1.06) | .451 |
| cVEMP 750 Hz | 0.98 (0.94-1.02) | .976 | 1.01 (0.97-1.05) | .529 |
| cVEMP 1000 Hz | 0.99 (0.95-1.03) | .643 | 0.99 (0.95-1.04) | .772 |
| OR = odds ration; CI = confidence interval | | | | |

| **Supplement Table 3: Correlation between audiometric testing and number of symptoms** | | | |
| --- | --- | --- | --- |
|  | **Number of Auditory Symptoms** | **Number of Vestibular Symptoms** | **Number of all symptoms** |
| **Preoperative Audiometric Testing** |  |  |  |
| ABG 250 Hz | R = .010 p = .911 | R = -.147 p= .110 | R = -.052 p= .582 |
| ABG 500 Hz | R = - .051 p= .587 | R =-.072  p = .442 | R = -.048 p = .612 |
| ABG 1000 Hz | R = - .022 p = .844 | R = -.082 p = .380 | R = -.023 p = .809 |
| ABG 2000 Hz | R = - .017p =.860 | R = -.191 p = .142 | R = -.074 p = .448 |
| ABG 4000 Hz | R = - .051 p= .586 | R = -.100 p = .287 | R = -.024 p =.803 |
| PTA | R = .050 p = .590 | R = -.068 p = .464 | R = -.010 p = .912 |
| cVEMP 500 Hz | R =-.013  p = .901 | R = .024 p = .814 | R = -.052 p = .618 |
| cVEMP 750 Hz | R = - .105 p = .328 | R = .018 p = .866 | R = -.071 p = .517 |
| cVEMP 1000 Hz | R = .028 p = .796 | R = .085 p = .425 | R = .060 p=.581 |
| Arc Length | R = .146 p = .164 | R = -.115 p = .274 | R = .059 p= .586 |
| Liner length | R = .175 p = .096 | R = -.065 p= .535 | R = .111 p =.305 |
| **Postoperative Audiometric Testing** |  |  |  |
| ABG 250 Hz | R = -.083 p= .263 | R = .075 p= .430 | R = -.049 p= .605 |
| ABG 500 Hz | R = -.015 p = .873 | R = -.009 p = .927 | R = -.040 p = .678 |
| ABG 1000 Hz | R = -.005  p = .949 | R = -.067 p = .482 | R = -.068 p = .480 |
| ABG 2000 Hz | R = .072 p =.439 | R = .045 p = .642 | R = .030 p = .759 |
| ABG 4000 Hz | R = -.059 p = .523 | R = -.048 p = .615 | R = -.103 p = .282 |
| PTA | R =  .082 =.377 | R = .015 p = .878 | R = .030 p = .757 |
| cVEMP 500 Hz | **R = -.229 p = .033** | R = .012 p = .913 | R = -.150 p = .180 |
| cVEMP 750 Hz | R = -.159 p = .141 | R = .063 p = .571 | R = -.085 p = .445 |
| cVEMP 1000 Hz | R = .-128 p = .240 | R = -.015 p = .892 | R = -.122 p=.278 |
| R indicates Pearson correlation coefficient. P-value obtained via 2-tailed significance | | | |
